# Robust SARS-CoV-2-specific and heterologous immune responses in vaccine-naïve residents of long-term care facilities who survive natural infection

**DOI:** 10.1038/s43587-022-00224-w

**Published:** 2022-05-30

**Authors:** Gokhan Tut, Tara Lancaster, Megan S. Butler, Panagiota Sylla, Eliska Spalkova, David Bone, Nayandeep Kaur, Christopher Bentley, Umayr Amin, Azar T. Jadir, Samuel Hulme, Morenike Ayodel, Alexander C. Dowell, Hayden Pearce, Jianmin Zuo, Sandra Margielewska-Davies, Kriti Verma, Samantha Nicol, Jusnara Begum, Elizabeth Jinks, Elif Tut, Rachel Bruton, Maria Krutikov, Madhumita Shrotri, Rebecca Giddings, Borscha Azmi, Chris Fuller, Aidan Irwin-Singer, Andrew Hayward, Andrew Copas, Laura Shallcross, Paul Moss

**Affiliations:** 1 Institute of Immunology and Immunotherapy, University of Birmingham, Birmingham, UK; 2 UCL Institute of Health Informatics, London, UK; 3 Department of Health and Social Care, London, UK; 4 Health Data Research UK, London, UK; 5 UCL Institute for Global Health, London, UK

**Keywords:** SARS-CoV-2, Infection, Ageing

## Abstract

We studied humoral and cellular immunity against severe acute respiratory syndrome coronavirus 2 (SARS-CoV-2) in 152 long-term care facility staff and 124 residents over a prospective 4-month period shortly after the first wave of infection in England. We show that residents of long-term care facilities developed high and stable levels of antibodies against spike protein and receptor-binding domain. Nucleocapsid-specific responses were also elevated but waned over time. Antibodies showed stable and equivalent levels of functional inhibition against spike-angiotensin-converting enzyme 2 binding in all age groups with comparable activity against viral variants of concern. SARS-CoV-2 seropositive donors showed high levels of antibodies to other beta-coronaviruses but serostatus did not impact humoral immunity to influenza or other respiratory syncytial viruses. SARS-CoV-2-specific cellular responses were similar across all ages but virus-specific populations showed elevated levels of activation in older donors. Thus, survivors of SARS-CoV-2 infection show a robust and stable immunity against the virus that does not negatively impact responses to other seasonal viruses.

## Abstract (web summary)

This study shows that during the first wave of SARS-CoV-2 infection in England, residents of long-term care facilities who survived infection developed a robust and stable immunity against the virus that did not negatively impact responses to other seasonal viruses.

## Main

A striking feature of the current SARS-CoV-2 pandemic has been the high rates of mortality in older people. The biological basis for this observation is unclear but may relate to relative impairment of innate immune or adaptive responses and increased prevalence of comorbidities. Long-term care facilities (LTCFs) provide residential and/or nursing care support for some of the frailest older adults (>65) in the population and as such have proven vulnerable to the impact of the SARS-CoV-2 pandemic^1^. Rates of SARS-CoV-2 infection in LTCFs have varied considerably and a range of factors have been determined that may impact susceptibility to infection^2^. Mortality rates among older adult residents have been estimated at up to 30% (ref. ^3^) with factors such as cognitive impairment and extreme aging as notable risk factors^3–5^. Nevertheless, most older people recover from acute SARS-CoV-2 infection and this is dependent on the generation of a functional SARS-CoV-2-specific immune response. This also helps to provide protection against subsequent reinfection and enhances immune responses at the time of coronavirus disease 2019 (COVID-19) vaccination. However, studies of staff and residents in LTCFs are difficult to perform and at the current time there is very limited information regarding the nature of the SARS-CoV-2-specific response after natural infection in this setting.

Vaccination against COVID-19 has proven highly effective; evidence to date shows that this protective effect is also observed in older adults and frail individuals^6,7^. As such, LTCF vaccination programs offer the potential to limit the impact of the pandemic in this setting. However, it is also important to determine how the features of natural immunity impact the immune response to vaccination as SARS-CoV-2 serostatus impacts on the response to both single and double vaccination. These studies are required to determine the relative need for booster vaccination, particularly in vulnerable populations where there has been concern about the longevity of vaccine-induced immunity.

A further issue of importance relates to the potential impact of SARS-CoV-2 serostatus on heterologous immune responses to other pathogens. Seasonal respiratory viruses such as influenza and respiratory syncytial virus (RSV) are a cause of considerable morbidity and mortality in older adults; it is currently unclear if primary SARS-CoV-2 infection, with its associated acute inflammatory response, might act to suppress memory antibody responses against other pathogens. Competition between plasma cells for space within the immunological niche of the bone marrow and their displacement by subsequent infections has been suggested to be a potential mechanism for loss of some antibody responses with aging^8^. If so, this could have a considerable impact on policy decisions in relation to the annual influenza vaccination strategy. Furthermore, there is increasing concern about the long-term complications of SARS-CoV-2 infection, which may partly reflect a sustained systemic inflammatory profile. This may be of particular importance in older people since many inflammatory markers increase naturally with age in a process that has been termed ‘inflamm-aging’ and is particularly marked in those with chronic health conditions^9^. To date, there is no information on how SARS-CoV-2 serostatus impacts systemic inflammatory markers in older people in the care home setting.

We determined virus-specific and general inflammatory profiles in both staff and residents in the LTCF setting over a 4-month period and related these findings to SARS-CoV-2 serostatus. Overall, robust SARS-CoV-2-specific and heterologous immune responses were observed across the life course, which is encouraging for longer-term health outcomes and responsiveness to COVID-19 vaccination.

## Results

### SARS-CoV-2-specific antibody responses are higher in residents

Blood samples were collected from 276 staff and residents at England LTCFs between June and November 2020, before the introduction of COVID-19 vaccination on 8 December 2020 (Table 1). Baseline samples were collected between June and July with matched follow-up samples at two and four months later. A total of 163 donors were SARS-CoV-2 seropositive based on nucleocapsid antibody status and 113 were seronegative. The peak time point for primary SARS-CoV-2 infection in LTCFs was April 2020; as such, these time points likely represent time points up to seven months after primary infection.

**Table 1:** Demographics of donors

Characteristic		
Total *n* participants	276	
Age in years, *n* (%)		
≥**80**	85 (31)	
65–80	46 (17)	
64–40	112 (40)	
≤**40**	33 (12)	
**Residents**	124 (45)	
**Staff**	152 (55)	
**Median age (IQR) of SARS-CoV-2 seropositive residents**	86 (76–90)	
**Median age (IQR) of SARS-CoV-2 seropositive staff**	57 (42–62)	
**Female,** ***n*** **(%)**	211 (76)	
**Male,** ***n*** **(%)**	65 (24)	
**Total** ***n*** **LTCFs**	38	
**Mean participants per LTCF (SD)**	7.2 (7.5)	
**Seropositive residents,** ***n*** **(%)**	70 (25)	
**Seronegative residents,** ***n*** **(%)**	53 (19)	
**Seropositive staff,** ***n*** **(%)**	93 (34)	
**Seronegative staff,** ***n*** **(%)**	60 (22)	

Initial studies focused on the assessment of SARS-CoV-2-specific humoral immunity in the 163 seropositive staff and residents. Meso Scale Discovery (MSD) technology was used to determine the magnitude of spike-specific, receptor-binding domain (RBD)-specific and nucleocapsid-specific antibody responses and data were compared between four age quintiles: <40; 40–64; 65–85; and 85+ years of age (*n* = 19, 67, 42 and 35, respectively; <65 years represents staff while >65 years represents residents). Serial analysis at two and four months after the initial collection allowed assessment of antibody waning during this period.

**Fig. 1:**
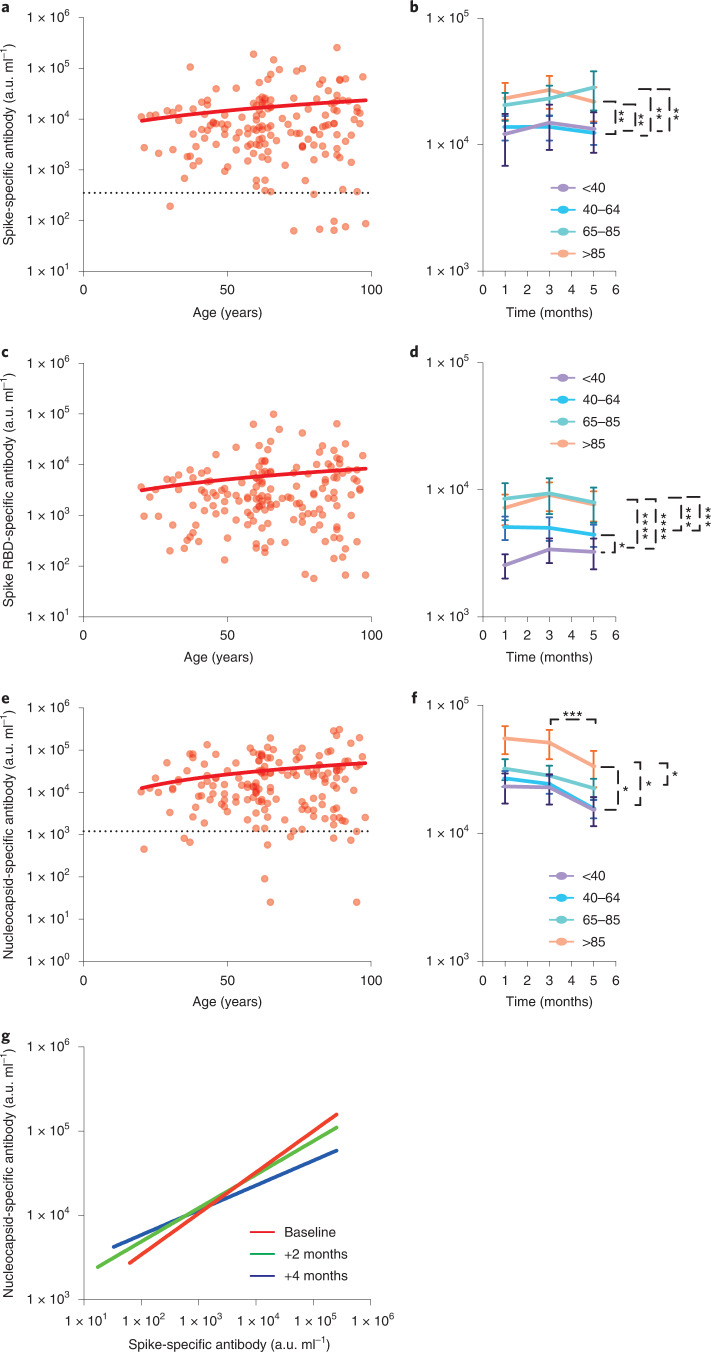
Elevated levels of SARS-CoV-2 spike and nucleocapsid-specific antibodies in LTCF residents. **a**, SARS-CoV-2 spike-specific IgG response in relation to age in seropositive donors (*n* = 163; two-tailed Pearson correlation coefficient *r* = 0.11, *P* = 0.16). The dotted black line indicates the cutoff. **b**, Mean spike-specific antibody responses at baseline and at the 2- and 4-month follow-up in 4 age groups: <40 (purple, *n* = 19); 40–64 (blue, *n* = 67); 65–85 (green, *n* = 42); and >85 years (orange, *n* = 35). The error bars indicate the s.e.m. Tukey’s multiple comparisons test, **P < 0.001. **c**, SARS-CoV-2 RBD-specific IgG response in relation to age in seropositive donors (*n* = 163) (two-tailed Pearson correlation coefficient *r* = 0.11, *P* = 0.17). **d**, Mean RBD-specific antibody responses at baseline and at the 2- and 4-month follow-up in 4 age groups: <40 (purple, *n* = 19), 40–64 (blue, *n* = 67), 65–85 (green, *n* = 42); and >85 years (orange, *n* = 35). The error bars indicate the s.e.m. Tukey’s multiple comparisons test, *P < 0.05, ***P = 0.0006, ****P < 0.0001. **e**, SARS-CoV-2 nucleocapsid-specific IgG response in relation to age in seropositive donors (*n* = 163; two-tailed Pearson correlation coefficient *r* = 0.19, *P* = 0.01). The dotted black line indicates the cutoff. **f**, Mean nucleocapsid-specific antibody responses at baseline and at the 2- and 4-month follow-up in 4 age groups: <40 (purple, *n* = 19); 40–64 (blue, *n* = 67), 65–85 (green, *n* = 42); and >85 years (orange, *n* = 35). The error bars indicate the s.e.m. Tukey’s multiple comparisons test, *P < 0.05, ***P = <0.01. **g**, Correlation between SARS-CoV-2 spike- and nucleocapsid-specific antibody responses in all donors at three time points, baseline (red) (two-tailed Pearson correlation coefficient *r* = 0.26), 2 months after baseline (green) (two-tailed Pearson correlation coefficient *r* = 0.17) and 4 months after baseline (blue) (two-tailed Pearson correlation coefficient *r* = 0.08) (*n* = 163 individuals with 3 sample collections each were tested). The lines of best fit are shown. All fitted lines were generated using a simple linear regression. a.u., arbitrary unit.

Spike-specific and RBD-specific antibody responses were both higher in LTCF residents aged 65+ years compared to younger donors with values that were increased by 1.7-times and 2.1-times, respectively (*P* = 0.004 and *P* ≤ 0.0001, respectively) (Fig. 1a–d). Nucleocapsid-specific responses were also increased in older adult donors aged 85+ years where they were 2.2-times higher than donors aged <65 years (*P* = 0.04) (Fig. 1e,f).

The potential importance of antibody waning was next assessed in relation to donor age. Temporal assessment of antibody responses over four months showed that antibody responses against SARS-CoV-2 spike and RBD were stable over time (Fig. 1g). This is encouraging in relation to potential protection against reinfection and supports clinical assessment in relation to the capacity of natural immunity to prevent infection.

In contrast, relative waning of antibody responses against nucleocapsid protein was observed in all age groups during the 4 months of study with average values falling by 34% during this time (Fig. 1e–g and Supplementary Table 3). This pattern of enhanced antibody waning against nucleocapsid has been seen in other settings^10^ and was reflected in spike:nucleocapsid antibody ratio in donors aged >85+ years, which decreased from 2.4-times at baseline to 1.6 times at 4 months of follow-up (*P* = 0.0003) (Fig. 1f and Supplementary Table 1).

### SARS-CoV-2-specific antibody responses are comparable to variants of concern

We next investigated the capacity of antibodies from both staff and residents to inhibit the binding of soluble recombinant spike protein to angiotensin-converting enzyme 2 (ACE2). Importantly, in addition to the prototypic Wuhan sequence, recombinant spike protein from the viral variants of concern (VOCs) B.1.1.7 (Alpha), B.351 (Beta) and P.1 (Gamma) was also incorporated in the assay.

**Fig. 2:**
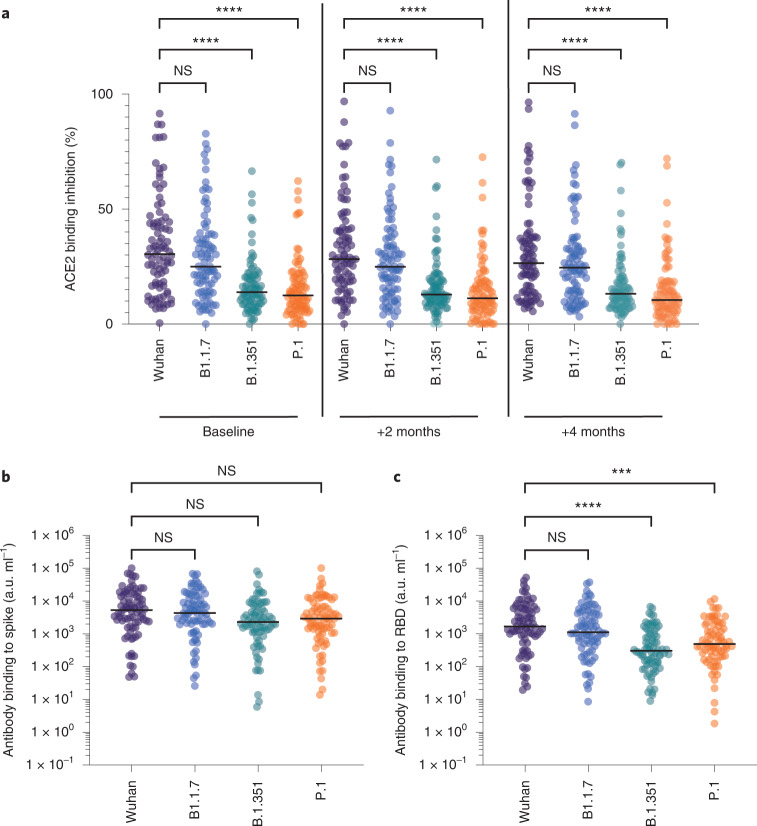
Serological inhibition of spike-ACE2 interaction and direct antibody binding to spike/RBD. **a**, Serological inhibition of spike binding to ACE2. Spike proteins were available from the viral variants Wuhan (purple), B1.1.7 (blue), B.1.351 (green) and P.1 (orange). Sera were obtained from donors after natural infection at baseline and then 2 and 4 months after the baseline sample. Tukey’s multiple comparisons test, ****P < 0.0001 (*n* = 163 individuals with 3 sample collections each were tested). **b**, Serological binding to SARS-CoV-2 spike from viral variants (Wuhan (purple), B1.1.7 (blue), B.1.351 (green) and P.1 (orange)) after natural infection at baseline. Tukey’s multiple comparisons test. NS, not significant (*n* = 163 individuals with 3 sample collections each were tested). **c**, Serological binding to SARS-CoV-2 RBD from viral variants (Wuhan (purple), B1.1.7 (blue), B1.351 (green) and P.1 (orange)) after natural infection at baseline. Tukey’s multiple comparisons test, ****P < 0.0001 (*n* = 163 individuals with 3 sample collections each were tested).

Immunosera showed markedly impaired inability to inhibit spike-ACE2 binding for VOCs compared to the parental Wuhan virus (34% s.d.: 21.7%). This was particularly true for the B.351 (16.6%, *P* ≤ 0.0001) and P.1 (15.4%, *P* ﻿≤ 0.0001) VOCs while inhibition of B.1.1.7 variant binding (29.4%, *P* = 0.78) was comparable to Wuhan in both assays (Fig. 2a). Of note, we also observed that this profile of VOC spike inhibition was stable over four months of follow-up, which indicates that the functional activity of antibodies does not decline (Fig. 2a). Furthermore, no differences were observed in relation to the pattern of spike inhibition using serum samples from donors across all ages (data not shown).

We next determined direct serum binding to spike proteins from VOCs. Of interest, antibody binding to total spike protein was comparable against all four VOCs indicating that the mutational changes in each VOC did not significantly change the total antibody binding capacity of the spike protein (Fig. 2b). However, serological neutralization of virus binding to ACE2 is mediated primarily through binding to the RBD; binding was substantially reduced against both B.351 (842 a.u. ml^−1^, *P* ﻿≤ 0.0001) and P.1 (1,377 a.u. ml^−1^, *P* = 0.0003) compared to 5,170 a.u. ml^−1^ against the Wuhan prototype (Fig. 2c). This profile was in line with the pattern of spike-ACE2 inhibition; again this pattern was both stable over time and across the age groups (data not shown).

These data show that the functional capacity of spike-specific antibodies elicited after natural infection is stable over at least four months and is independent of age in the LTCF setting.

### SARS-CoV-2 infection boosts antibody responses against beta-coronaviruses

We next went on to assess how previous infection with SARS-CoV-2 impacted on the magnitude of the antibody response against other human coronaviruses. The MSD platform was used to assess binding to spike protein from the beta-coronaviruses SARS-CoV-1, HKU1 and OC43 as well as the alpha-coronaviruses 229E and NL63.

**Fig. 3:**
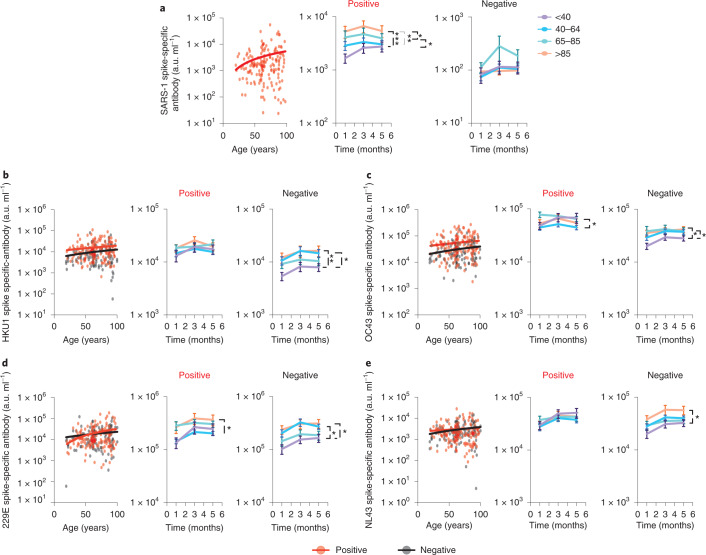
IgG antibody responses against beta-coronaviruses are increased in donors with previous SARS-CoV-2 infection. Comparison of antibody responses to coronaviruses in relation to age and SARS-CoV-2 serostatus. Seronegative donors are indicated by black dots (*n* = 113) and seropositive donors (*n* = 163) are indicated by red dots. The lines indicate the line of best fit. Mean responses over 4 months of follow-up are shown in 4 age ranges: <40 (purple); 40–64 (blue); 65–85 (green); and >85 years (orange). The error bars indicate the s.e.m. **a**, SARS-1 spike-specific antibody response in SARS-CoV-2 seropositive donors (two-tailed Pearson correlation coefficient *r* = 0.16, *P* = 0.03). Right: Stability of response in the age cohorts. Tukey’s multiple comparisons test, *P = 0.035, **P = 0.0013, ***P = 0.0003. **b**, HKU1 spike-specific antibody response (seropositive: two-tailed Pearson correlation coefficient *r* = 0.10, *P* = 0.16; seronegative: two-tailed Pearson correlation coefficient *r* = 0.14, *P* = 0.13). Right: Stability of response in the age cohorts. Tukey’s multiple comparisons test, *P = 0.01, **P = 0.008. **c**, OC43 spike-specific antibody response (seropositive: two-tailed Pearson correlation coefficient *r* = 0.11, *P* = 0.15; seronegative: two-tailed Pearson correlation coefficient *r* = 0.17, *P* = 0.06). Right: Stability of response in the age cohorts. Tukey’s multiple comparisons test: seropositive *P = 0.01; seronegative *P ﻿≤ 0.02. **d**, 229E spike-specific antibody response (seropositive: two-tailed Pearson correlation coefficient *r* = 0.23, *P* = 0.003; seronegative: two-tailed Pearson correlation coefficient *r* = 0.13, *P* = 0.16). Right: Stability of response in age cohorts. Tukey’s multiple comparisons test: seropositive *P* = 0.02; seronegative *P ﻿≤ 0.03. **e**, NL63 spike-specific antibody response (seropositive: two-tailed Pearson correlation coefficient *r* = 0.06, *P* = 0.42; seronegative: two-tailed Pearson correlation coefficient *r* = 0.19, *P* = 0.03). Right: Stability of response in the age cohorts. Tukey’s multiple comparisons test seronegative *P ﻿≤ 0.02. All fitted lines were generated using a simple linear regression.

As anticipated, antibody binding to SARS-CoV-1 was very low in SARS-CoV-2-seronegative individuals but substantially increased in seropositive donors^11^ due to substantial sequence homology between viruses. This was strongly enhanced in older donors reflecting the elevated SARS-CoV-2-specific response in this age group (*r* = 0.16, *P* = 0.03) (Fig. 3a).

However, it was also noteworthy that enhanced antibody binding was observed against both of the endemic beta-coronaviruses in SARS-CoV-2-positive donors (OC43 55,451 a.u. ml^−1^ versus 31,539 a.u. ml^−1^, *P* ﻿≤ 0.0001 and HKU1 16,223 a.u. ml^−1^ versus 10,083 a.u. ml^−1^, *P* = 0.0008) (Fig. 3b,c). Of note, OC43-specific responses were also enhanced in seronegative older people (*r* = 0.17, *P* = 0.06), potentially reflecting the impact of recurrent priming from seasonal infection (Fig. 3b,c).

Alpha-coronaviruses show less structural homology with SARS-CoV-2 spike protein and this may underlie the finding of no increment in serological binding to spike protein in SARS-CoV-2 seropositive donors. However, the serological response to alpha-coronaviruses was again increased in older people before SARS-CoV-2 infection (NL63: *r* = 0.19, *P* = 0.03) but this differential was lost after seroconversion (Fig. 3d,e).

These findings show that serological responses against both alpha- and beta-coronaviruses increase with age, potentially due to recurrent infections. However, cross-reactive antibody responses are increased after SARS-CoV-2 infection and this effect is both stronger in younger people and more marked for responses against beta-coronaviruses^12,13^. The relationship of these observations to clinical protection against individual coronaviruses is uncertain at present.

### SARS-CoV-2 serostatus does not impair antibody responses against other respiratory viruses

Acute respiratory infections such as influenza and RSV represent a major and continuous challenge to the health of older individuals; annual vaccination against influenza is recommended in many countries. Protection against infection is mediated primarily through the action of humoral immunity and there is concern that SARS-CoV-2 serostatus may impact on the titer of heterologous immune responses. As such, we next assessed the strength of antibody responses against a range of influenza viral strains in relation to age and SARS-CoV-2 serostatus.

**Fig. 4:**
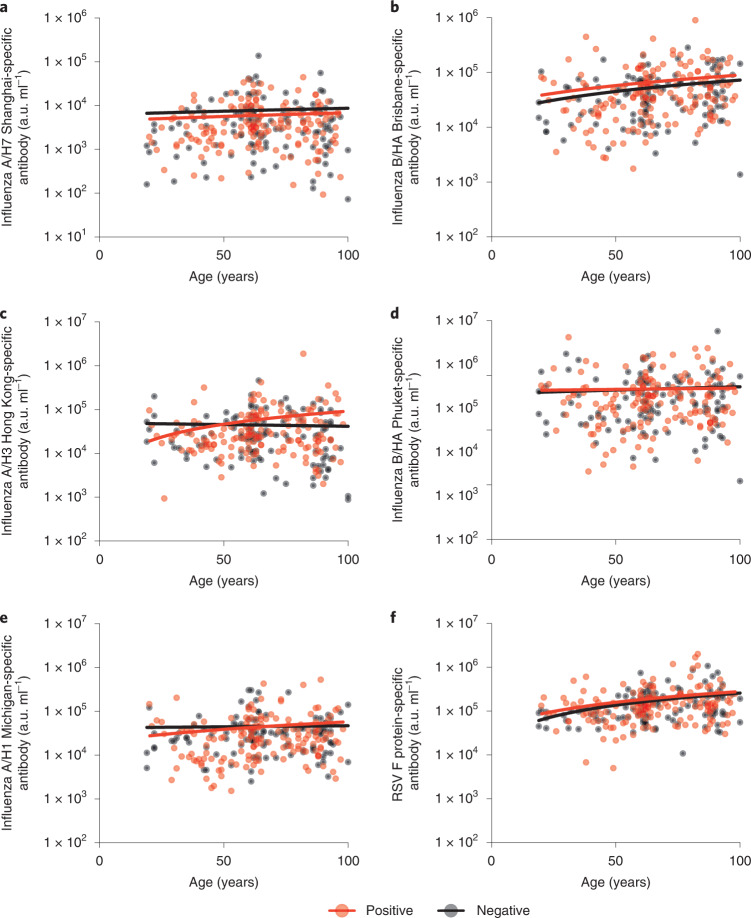
SARS-CoV-2 serostatus does not influence IgG responses to other respiratory viruses. Comparison of IgG responses against respiratory viruses in relation to age and SARS-CoV-2 serostatus. The red dots indicate positive donors (*n* = 164) and the black dots indicate negative donors (*n* = 113). Statistical analysis is shown for seropositive and seronegative donors, respectively. **a**, Influenza A/H7 Shanghai (two-tailed Pearson correlation coefficient *r* = 0.06, *P* = 0.40; *r* = 0.03, *P* = 0.72). **b**, Influenza B/HA Brisbane (two-tailed Pearson correlation coefficient *r* = 0.12, *P* = 0.10; *r* = 0.22, *P* = 0.15). **c**, Influenza A/H3 Hong Kong (two-tailed Pearson correlation coefficient *r* = 0.11, *P* = 0.16; *r* = −0.02, *P* = 0.77). **d**, Influenza B/HA Phuket (two-tailed Pearson correlation coefficient *r* = 0.18, *P* = 0.81; *r* = −0.04, *P* = 0.64). **e**, Influenza A/H1 Michigan (two-tailed Pearson correlation coefficient *r* = 0.10, *P* = 0.18; *r* = −0.01, *P* = 0.85). **f**, RSV fusion protein (two-tailed Pearson correlation coefficient *r* = 0.18, *P* = 0.02; *r* = −0.29, *P* = 0.001). All fitted lines were generated using a simple linear regression.

SARS-CoV-2 serostatus did not have any influence on the titer of influenza or RSV-specific antibody responses across the different age groups (Fig. 4). Relative antibody titers against influenza were broadly stable across the life course while the serological response against RSV increased with age.

These data provide reassurance that previous history of SARS-CoV-2 infection does not impact detrimentally on the profile of immunity against heterologous respiratory viruses such as influenza or RSV.

### SARS-CoV-2-specific cellular responses are similar across all ages

Cellular immune responses are likely to play an important role in protection against reinfection with SARS-CoV-2. As such, interferon-γ (IFN-γ) enzyme-linked immunosorbent spot (ELISpot) analysis was next used to determine T cell responses against both a peptide pool from spike protein and a combination of peptides from nucleocapsid, membrane and envelope protein (N/M/E). Analysis was performed in 80 donors and assessed in 3 age groups: <40; 40–64; and 65+ years. The <40 and 40–64 age groups represent staff while the 65+ age group comprises LTCF residents.

**Fig. 5:**
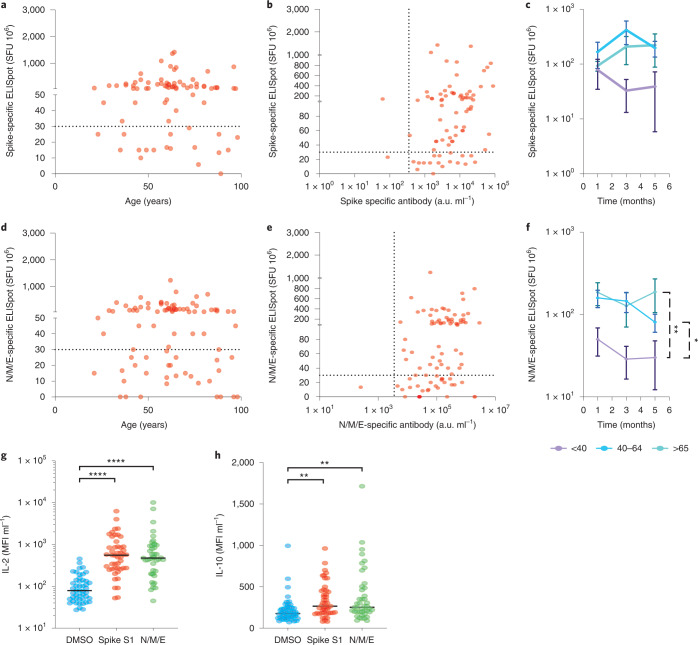
SARS-CoV-2-specific T cell responses are observed across the life course and demonstrate IFN-γ, IL-2 and IL-10 production. **a**, Spike-specific cellular response in relation to age in seropositive donors (*n* = 80) (two-tailed Pearson correlation coefficient *r* = 0.02, *P* = 0.82). The dotted black line indicates the cutoff for a positive response. **b**, Comparison of spike-specific cellular and antibody responses in seropositive donors (*n* = 80) (two-tailed Pearson correlation coefficient *r* = 0.32, *P* = 0.003). **c**, Mean spike-specific cellular responses in the age categories <40 (purple), 40–64 (blue) and 65+ years (green) at 0, 2 and 4 months. The error bars indicate the s.e. Tukey’s multiple comparisons test showed no significance. **d**, N/M/E-specific cellular response in relation to age in seropositive donors (*n* = 80) (two-tailed Pearson correlation coefficient *r* = −0.03, *P* = 0.72). The dotted black line indicates the cutoff for a positive response. **e**, Comparison of N/M/E-specific cellular and antibody responses in seropositive donors (*n* = 80) (two-tailed Pearson correlation coefficient *r* = 0.35, *P* = 0.001). **f**, Mean N/M/E-specific cellular responses in the age categories <40 (purple), 40–64 (blue) and >65 years (green) at 0, 2 and 4 months. The error bars indicate the s.e. Tukey’s multiple comparisons test, *P = 0.02, **P = 0.005. **g**, IL-2 concentration in ELISpot supernatants in DMSO (blue), spike S1 (red) and N/M/E (green) peptide-stimulated wells (*n* = 47 individuals tested from the ELISpot supernatants). Tukey’s multiple comparisons test, ****P ≤ 0.0001. **h**, IL-10 concentration in ELISpot supernatants in DMSO (blue), spike S1 (red) and N/M/E (green) peptide-stimulated wells (*n* = 47 individuals tested from the ELISpot supernatants). Tukey’s multiple comparisons test, **P ﻿≤ 0.002

The magnitude of the ELISpot response against spike peptides was comparable in donors at all ages and was also stable over the four months of follow-up (Fig. 5a–c). In contrast, cellular responses against the N/M/E pool were lower in donors aged under 40 years (36 spot-forming units (SFU) 10^6^) when compared to the 40–64 (129 SFU 10^6^, *P* = 0.02) and 65+ age groups (167 SFU 10^6^, *P* = 0.005) (Fig. 5d–f).

**Extended Data Fig. 1:**
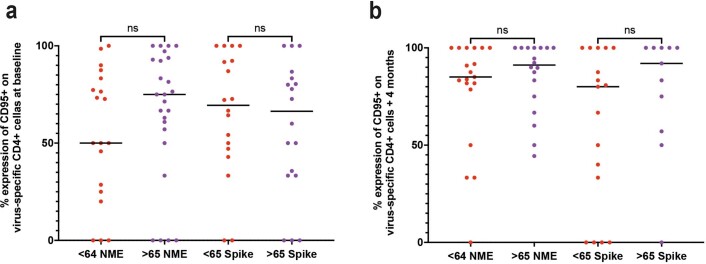
IL-10 and IL-2 concentration in ELISpot supernatants at 4 month timepoint and in relation to age. **a**) IL-2 concentration in ELISpot supernatants in DMSO negative control (blue), S1 (red) and N/M/E (green) peptide-stimulated wells (n = 47 individuals tested from ELISpot supernatants). Two tailed Pearson correlation no statistical significance seen. **b**) IL-10 concentration in ELISpot supernatants in DMSO negative control (blue), S1 (red) and N/M/E (green) peptide-simulated wells (n = 47 individuals tested from ELISpot supernatants). Two tailed Pearson correlation no statistical significance seen. All fitted lines are generated using a simple linear regression.

To further assess the functional profile of SARS-CoV-2-specific cells, we measured the concentration of cytokines in ELISpot eluates using the LEGENDplex technology. Markedly increased concentrations of interleukin-2 (IL-2) and interleukin-10 (IL-10) were seen in both spike- and N/M/E-specific eluates compared to the dimethyl sulfoxide (DMSO) control (Fig. 5g,h). Spike: IL-2; 110 mean fluorescence intensity (MFI) units ml^−1^ (control) versus 890 MFI ml^−1^, *P* ﻿≤ 0.0001; IL-10: 220 MFI ml^−1^ (control) versus 330 MFI ml^−1^, *P* = 0.002; N/M/E: IL-2; 110 MFI ml^−1^ (control) versus 1,000 MFI ml^−1^, *P* ﻿≤ 0.0001; IL-10: 215 MFI ml^−1^ (control) versus 380 MFI ml^−1^, *P* = 0.0019). No association with age was seen (Extended Data Fig. 1).

### SARS-CoV-2-specific CD4^+^ T cells are present at low frequency

**Fig. 6:**
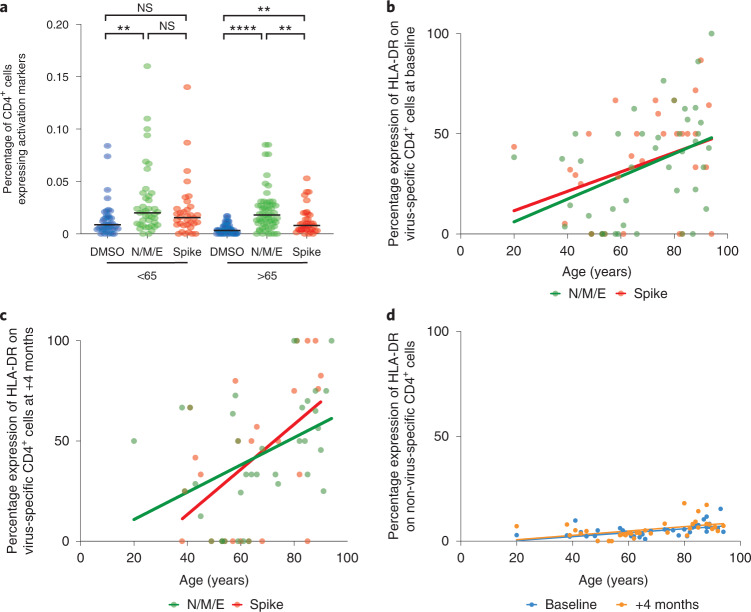
SARS-CoV-2-specific T cell response in staff and residents in LTCFs. **a**, Quantification of SARS-CoV-2-specific T cells based on percentage expression of both CD137^+^ and CD154^+^ on CD4^+^ cells after stimulation with N/M/E or spike peptides. DMSO was used as the negative control. Analysis is grouped into staff (<65 years) or residents (>65 years). The black lines indicate the median. Tukey’s multiple comparisons test, **P ≥ 0.002, ****P ≤ 0.0001. **b**,**c**, Percentage expression of HLA-DR^+^ on CD137^+^CD154^+^CD4^+^ cells after stimulation with N/M/E (two-tailed Pearson correlation coefficient *r* = 0.40, *P* = 0.006; *n* = 44) or spike peptides (two-tailed Pearson correlation coefficient *r* = 0.36, *P* = 0.03; n = 33) in relation to age at baseline (**b**) and at 4 months (**c**). **d**, Percentage expression of HLA-DR on bulk unstimulated CD4^+^ cells in relation to age at baseline (two-tailed Pearson correlation coefficient *r* = 0.63, *P* ﻿≤ 0.0001; *n* = 47) and at the 4-month follow-up (two-tailed Pearson correlation coefficient *r* = 0.54, *P* = 0.0005; *n* = 38). All fitted lines were generated using a simple linear regression.

**Extended Data Fig. 2:**
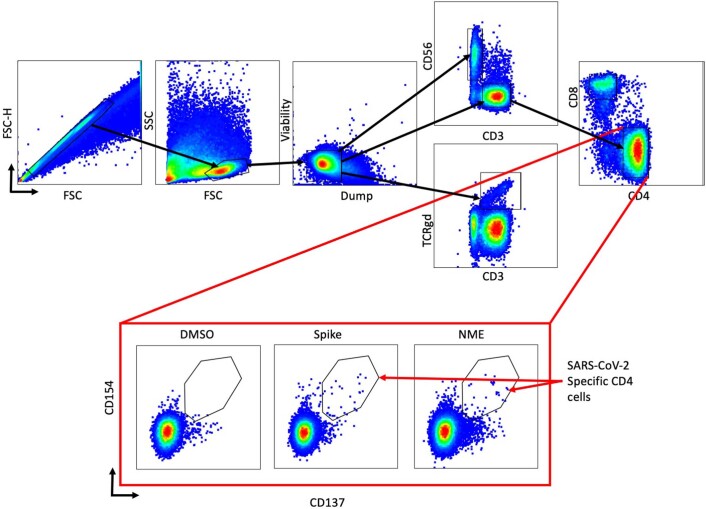
AIM gating strategy to isolate SARS-CoV-2 specific CD4 cells.

Activation-induced marker assessment was used to determine the magnitude and profile of the SARS-CoV-2-specific immune response after stimulation with viral peptide pools (*n* = 44) (Extended Data Fig. 2). The magnitude of the SARS-CoV-2-specific T cell response was modest and comparable across age groups (Fig. 6a). In particular, in LTCF staff a median of 0.02% and 0.016% of CD4^+^ cells demonstrated specificity for N/M/E or spike, respectively. Comparable values in the resident population were 0.018% and 0.008%, respectively. (Fig. 6a), which is comparable with previous reports. Virus-specific CD8^+^ cells were not detected with this approach^14^.

**Extended Data Fig. 3:**
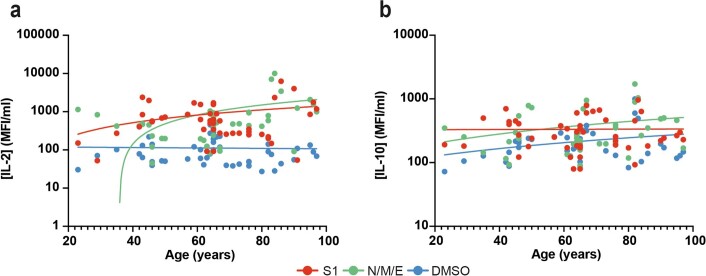
MFI of HLA-DR expression on virus-specific CD4+ cells in relation to age from spike-specific (Two tailed Pearson correlation r = 0.21 p = 0.34; n = 22) and NME-specific cells (Two tailed Pearson correlation r = 0.23 p = 0.14; n = 42). MFI of HLA-DR expression on virus-specific CD4+ cells in relation to age from spike-specific (Two tailed Pearson correlation r = 0.21 p = 0.34; n = 22) and NME-specific cells (Two tailed Pearson correlation r = 0.23 p = 0.14; n = 42).

**Extended Data Fig. 4:**
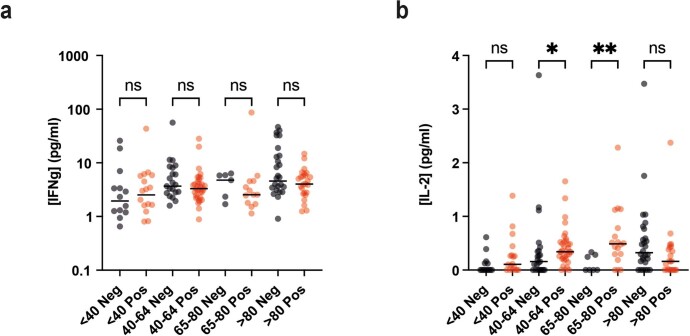
Percentage of CD95+ expression on SARS-CoV-2-specific CD4+ cells at baseline and at 4 months follow up in donors aged <65 or 65 + years. **a**, **b**) Quantification of CD95+ on CD4+ virus-specific cells after stimulation with N/M/E or spike peptides. Red dots are donors aged <65 years (n = 19) and purple dots are donors aged >65 years(n = 25). Tukey’s multiple comparations test, no significance seen.

Expression of memory and differentiation markers on the virus-specific pool demonstrated a dominant CD27^+^CD28^+^CD127^low^ CD4^+^ effector phenotype (data not shown). Of note, expression of the activation marker human leukocyte antigen DR isotype (HLA-DR) increased substantially with age on both spike- and N/M/E-specific CD4^+^ T cells although MFI was stable (spike: *r* = 0.36, *P* = 0.03, N/M/E: *r* = 0.40, *P* = 0.006) (Fig. 6b,c and Extended Data Fig. 3). A modest increase of HLA-DR expression was also seen on bulk CD4^+^ cells in relation to age but this was markedly lower than seen on virus-specific cells (Fig. 6d). This profile was present at baseline and was further accentuated on the spike-specific pool at the 4-month time point where a median of 75% of cells in residents expressed HLA-DR compared to 25% of cells in staff (*n* = 28; *P* = 0.02) (Fig. 6b,c). CD95 (Fas) expression was similar but increased during follow-up (Extended Data Fig. 4). Expression of CD25, an additional marker of activation, did not show any such relationship with age (data not shown).

These data indicate that SARS-CoV-2-specific T cells comprise a small component of the peripheral T cell pool but virus-specific cells in older people showed increased expression of HLA-DR suggesting activation.

### Serum inflammatory cytokine levels increase with age

Acute primary infection with SARS-CoV-2 increases serum concentration of many inflammatory cytokines such as interleukin-6 (IL-6), tumor necrosis factor-α (TNF-α) and IFN-γ. Sustained elevated levels of these proteins have been seen in a subgroup of patients with more prolonged disease. Thus we next assessed systemic cytokine concentrations in relation to SARS-CoV-2 serostatus across the life course.

**Fig. 7:**
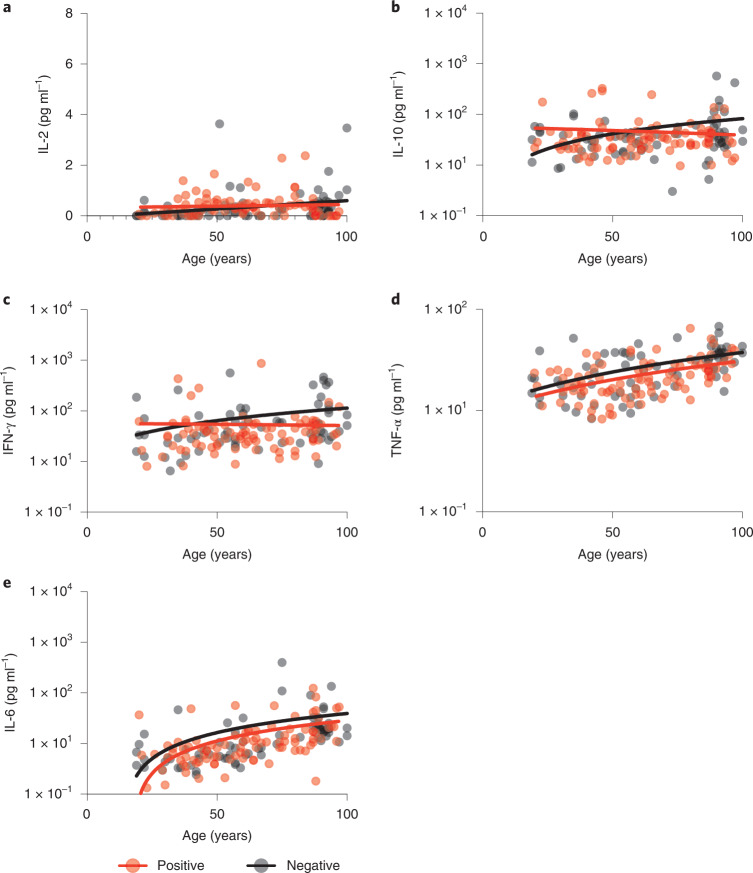
Plasma cytokine concentrations in relation to age and SARS-CoV-2 serostatus. **a**, IL-2 (two-tailed Pearson correlation coefficient *r* = 0.41, *P* = 0.0003; *r* = 0.008, *P* = 0.93). **b**, IL-10 (two-tailed Pearson correlation coefficient *r* = 0.20, *P* = 0.08; *r* = 0.11, *P* = 0.28). **c**, IFN-γ (two-tailed Pearson correlation coefficient *r* = 0.46, *P* ≤ 0.0001; *r* = 0.18, *P* = 0.07). **d**, TNF-α (two-tailed Pearson correlation coefficient *r* = 0.52, *P* ≤ 0.0001; *r* = 0.47, *P* ≤ 0.0001). **e**, IL-6 (two-tailed Pearson correlation coefficient *r* = 0.67, *P* ≤ 0.0001; *r* = 0.59, *P* ≤ 0.0001). The black dots indicate seronegative donors (*n* = 72) and the red dots indicate seropositive donors (*n* = 93). Statistical analysis assessed concentration in relation to age and is shown for seronegative and seropositive donors, respectively. All fitted lines were generated using a simple linear regression.

**Extended Data Fig. 5:**
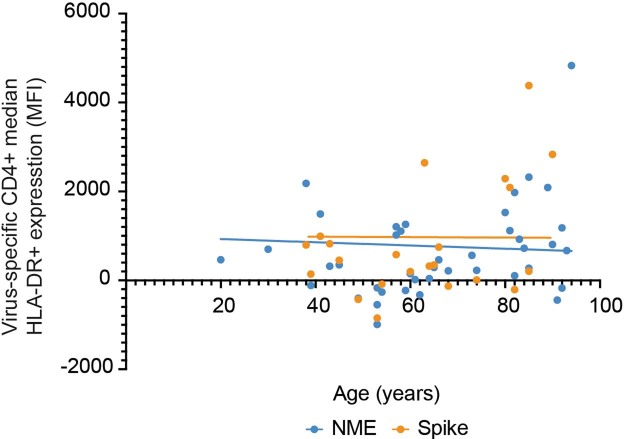
Plasma IFN-γ and IL-2 concentration in relation to SARS-CoV-2 serostatus and age quartile. **a**) IFN-γ concentration from SARS-CoV-2 seropositive (red, n = 92) and seronegative (black, n = 72) donors. Tukey’s multiple comparations test no significance seen. **b**) IL-2 concentration from SARS-CoV-2 seropositive (red, n = 92) and seronegative (black, n = 72) Tukey’s multiple comparations test * p = 0.02 **p = 0.005.

MSD analysis was used to measure the concentrations of IFN-γ, TNF-α, IL-6, IL-2 and IL-10 in plasma samples (Fig. 7 and Extended Data Fig. 5). Concentrations of the inflammatory cytokines IL-6, IFN-γ, IL-2 and TNF-α increased with age in SARS-CoV-2 seronegative donors and likely reflect the established profile of ‘inflamm-aging’^9^. No further increase with age was seen in SARS-CoV-2 seropositive individuals. As such these findings indicate that previous infection with SARS-CoV-2 does not appear to enhance systemic inflammation of inflamm-aging.

## Discussion

The SARS-CoV-2 pandemic has presented a major challenge to the management of LTCFs with high rates of mortality among older and frail residents. Little is known about the profile of SARS-CoV-2-specific immunity or the potential impact of previous infection on heterologous immunity. In this study, we investigated SARS-CoV-2-specific immune responses in staff and residents of LTCFs and found robust responses against spike protein across all age groups and no negative impact on immunity to other respiratory viruses. These findings are broadly reassuring for future management decisions.

Antibody responses against the SARS-CoV-2 spike protein and RBD are thought to be critical in the prevention against reinfection. As such we were encouraged to find that antibody responses against spike and RBD were robust in older people. Indeed, antibody titers were increased in people over 65 years of age. Nucleocapsid-specific antibody responses were also higher in LTCF residents. The reasons for this are not clear but may potentially reflect an elevated residual antibody ‘set point’ after enhanced response to higher levels of viral load at the time of primary infection in older individuals. It is also interesting to speculate on the potential relative ongoing exposure to virus for residents and staff although there are no data at present to suggest that this differs markedly. Nevertheless, these findings are in line with the 85% protection against reinfection that is seen in the resident population, a level of protection somewhat higher than the value of 60% in staff^15,16^.

Antibody responses against human endogenous alpha- and beta-coronaviruses show marked waning within the first year after infection and this has led to concern that a similar process may occur after SARS-CoV-2 infection^17^. Reassuringly, we observed that antibody responses against both spike and RBD were well maintained for at least four months. These data are in line with reports showing stability of spike-specific responses in younger donors^18^. In contrast, antibody responses against nucleocapsid protein showed significant waning in all age groups and represent a potential future challenge for the identification of individuals with prior natural infection. The physiological importance of the nucleocapsid-specific antibody response in relation to protection against reinfection is somewhat unclear. This profile of differential waning of nucleocapsid- and spike-specific antibody responses has been observed in other cohorts^19^ and may reflect alternative mechanisms of antigen presentation at the time of primary infection, with spike protein dominant on the viral envelope and nucleocapsid present in high numbers within the virion. Furthermore, retention of spike protein has been demonstrated within the gastrointestinal tract and may serve to drive maintenance and somatic hypermutation of spike-specific humoral responses^20^.

We were also interested in assessing the relative functional activity of antibodies in relation to age. Again, this was comparable in both staff and residents with comparable inhibition of binding of Wuhan virus spike protein to the ACE2 receptor. Inhibition was also assessed against SARS-COV-2 viral VOCs where reduced activity was seen against both the B.1.351 and P.1 variants, in line with previous reports^21^, but was equivalent in staff and residents. Furthermore, binding inhibition remained stable over four months, indicating maintenance of antibody avidity. It is worth mentioning that seropositive participants were infected in the first wave of the pandemic before any known VOCs; therefore, it is most likely that they were infected with the Wuhan strain.

Infection with heterologous viruses such as influenza and RSV is a major health concern for LTCF residents and there is currently no information on how SARS-CoV-2 serostatus may impact on memory responses against these respiratory viruses. This is of note since during the pandemic there has been a low incidence of non-COVID respiratory infections due to social distancing and there is concern that rapid reemergence of influenza and RSV infections may emerge as LTCFs start to reduce use of disease control measures. Infection with viruses such as measles can erode antibody responses against other pathogens^22,23^, potentially due to displacement of plasma cells within the bone marrow. Again, it was encouraging to see that the breadth and magnitude of the human response against influenza subtypes were entirely comparable across donors irrespective of SARS-CoV-2 infection status. This provides reassurance that residents who have undergone SARS-CoV-2 infection will not show increased vulnerability against seasonal respiratory infections. It will now be of interest to assess the relative response after annual influenza vaccination.

The importance of cellular immunity in relation to the protection against SARS-CoV-2 reinfection is uncertain but it is likely that cellular responses play a critical role in protection against severe disease^24^. Impairment of natural and vaccine-induced cellular immune responses is a feature of immune senescence and it was therefore noteworthy that spike-specific cellular responses were of similar magnitude in both staff and residents. Indeed, cellular responses against nucleocapsid protein were increased in donors aged over 40 years and therefore mirrored the increased profile of humoral immunity against this protein in relation to age. Functional activity was demonstrated by IFN-γ production within the ELISpot assay while IL-2 and IL-10 were also produced by virus-specific T cells. IL-2 production is a characteristic feature of the SARS-CoV-2 cellular response and suggests that the SARS-CoV-2-specific cellular response resides within a moderately differentiated effector pool but is not driven to terminal differentiation. Less is known about the role of the immunosuppressive cytokine IL-10. Cellular responses against both the spike and nucleocapsid proteins were stable over the four months of follow-up and again concur with data from younger donors^14,25^.

Phenotyping of virus-specific CD4^+^ T cells indicated a moderately differentiated CD27^+^CD28^+^ phenotype, which is consistent with the predominant IL-2 cytokine phenotype. It was notable that expression of the HLA-DR activation marker was markedly increased on T cells from LTCF residents and this increased further over time. This suggests potential ongoing activation of virus-specific cells in older people, possibly due to increased retention of viral antigen; this will be important to assess in longer-term follow-up.

Finally, we also measured the serum concentration of several cytokines across the life course and related these to SARS-CoV-2 serostatus. Ongoing clinical data were not available from the cohort and as such we were unable to relate these values to health outcomes. An increase in IL-6 and TNF-α concentration with age was apparent and is widely recognized as a marker of ‘inflamm-aging’^26^. Importantly, however, viral serostatus did not have any influence on the magnitude of this trajectory indicating that history of previous infection does not accelerate inflammation in this age group.

Vaccination has emerged as a highly effective approach to elicit protection against severe COVID-19 infection^7^. As such it is important to interpret these findings in relation to vaccine responses in this population. Previous natural infection with SARS-CoV-2 strongly enhances spike-specific immune responses after vaccination and our findings likely underlie the 23-fold elevation of peak antibody responses after single vaccination in SARS-CoV-2 seropositive LTCF residents^27^. These data indicate that short-term vaccine responses in LTCF residents are likely to be comparable to those of younger people. It will be critical to assess if the comparable maintenance of immunity after natural infection translates into a similar profile after vaccination.

It is interesting to reflect on these findings in relation to our current understanding of the mechanisms of immune senescence that often limit the quality of adaptive immune responses against pathogens. These are thought to include attrition of the naïve lymphoid pool, accumulation of senescent cells and increased levels of inflammatory mediators. Our findings that the magnitude and functional quality of adaptive responses against SARS-CoV-2 are robust in older people that survive SARS-COV-2 infection is therefore of some interest. It is not likely that cross-reactive adaptive responses against seasonal human coronaviruses contribute significantly to SAR-CoV-2 responses in older people; as such de novo B and T cell clonal responses would be expected to be recruited from the naïve repertoire. Whether or not germline antigen receptors with specificity for coronaviruses have been selected during evolution and may therefore provide a framework for development of high-affinity SARS-CoV-2-specific responses are unclear although the application of prospective antigen receptor sequencing across age groups will be of interest.

Potential limitations of our study include the fact that all seropositive donors clearly represent survivors of acute SARS-CoV-2 infection and, since mortality rates were high in the LTCF resident age group, there may have been potential selection bias for donors with the most effective underlying immune function. Additionally, we did not have access to the exact time or severity of primary infection or history of patient comorbidities. Sex did not impact on immune response but information on participant ethnicity was not available.

These findings indicate that immune responses to SARS-CoV-2 infection in older adults and potentially frail resident LTCF population are robust and comparable to those seen in younger people. These findings are consistent with similar levels of protection against reinfection in this cohort and provide insight into the potent influence of previous infection on the magnitude of the immune response to COVID-19 vaccination. It is now important to assess how infection status acts to support the longevity of vaccine-induced immune responses and if this should be used as a determinant of the need for multiple vaccine boosters.

## Methods

### Sample collection

The VIVALDI study (ISRCTN14447421) (ongoing) is a prospective cohort study set up in May 2020 to investigate SARS-CoV-2 transmission, infection outcomes and immunity in residents and staff in LTCFs in England that provide residential and/or nursing care for adults aged 65 years and older (https://wellcomeopenresearch.org/articles/5-232/v2 (ref. ^28^)). Eligible LTCFs were identified by the Care Provider’s Senior Management Team or by the National Institute for Health Research (NIHR) Clinical Research Network. Pseudonymized clinical (vaccination status, PCR test results) and demographic (age, sex, staff member versus resident) data were retrieved for staff and residents from participating LTCFs through national surveillance systems. All participants provided written informed consent for blood sample collection; if residents lacked the capacity to consent, a personal or nominated consultee was identified to act on their behalf.

Blood sampling was offered to participants at three time points in June–July, August–September and October–November 2020. This time period was before the national vaccine rollout; therefore, all data assess the immune response to natural infection alone. The anticoagulated (EDTA) sample was sent to the University of Birmingham and the serum tube to The Doctors Laboratory for anti-nucleocapsid IgG testing. Ethical approval for this study was obtained from the South Central-Hampshire B Research Ethics Committee (ref. no. 20/SC/0238).

### Data linkage

Abbott antibody test results were submitted to the COVID-19 Data Store Reference Library (https://data.england.nhs.uk/covid-19/) and linked to routinely held data on age, sex, LTCF and role (staff or resident) obtained through the national SARS-CoV-2 testing program and vaccination status (date and vaccine type) derived from the National Immunisations Management System. These records were linked using a common identifier based on the individual’s NHS number. Individual-level records were further linked to each LTCF using the unique Care Quality Commission location ID, allocated by the Care Quality Commission who regulate all providers of health and social care in the UK.

### Inclusion criteria

Both staff and residents were eligible for inclusion if it was possible to link them to a pseudo-identifier in the COVID-19 Data Store. Only those who had been infected in the first wave of the pandemic in the UK or those who had no evidence of being infected were included in this subcohort. Samples from LTCFs that had experienced outbreaks in the first wave were chosen for seropositive samples and LTCFs with no outbreaks were chosen for the seronegative samples. Participants who seroconverted during the four-month follow-up were not included in the analysis. Due to limited PCR testing in the first wave of the pandemic, it was not possible to determine the date of primary infection with SARS-CoV-2; 21 April 2020 was the peak of the first wave for SARS-CoV-2 infection in LTCFs. Past infection with SARS-CoV-2 was defined based on results of Abbott antibody and MSD quantitative test using the thresholds and methods outlined below.

### Sample preparation

Samples were processed within 24 h of reception at the University of Birmingham. Blood was spun at 300 *g* for 5 min. Plasma was removed and spun at 500 *g* for 10 min before storage at −80 °C. The remaining blood was separated using a SepMate (STEMCELL Technologies) density centrifugation tube. The resulting peripheral blood mononuclear cell (PBMC) layer was washed twice with Roswell Park Memorial Institute (RPMI) 1640 medium and frozen in freezing medium (10% DMSO, 90% FCS).

### Serological analysis of SARS-CoV-2-specific immune response

Quantitative IgG antibody titers were measured against trimeric spike protein. Multiplex MSD assays were performed according to the manufacturer’s instructions (panels 7 and 13, lot no. K0081681). Briefly, 96-well plates were blocked. After washing, samples were diluted 1:5,000 in diluent and added to the wells with the reference standard and internal controls. After incubation, plates were washed and anti-IgG detection antibodies were added. Plates were washed and were immediately read using a MESO TM QuickPlex SQ 120 system. Data were generated by the Methodological Mind v1.0 software and analyzed with the MSD Discovery Workbench v.4.0 software. The presented data were adjusted for any sample dilution.

To assess serological response against the SARS-CoV-2 nucleocapsid, blood samples were tested for the presence of IgG antibodies specific for nucleocapsid protein using the Abbott ARCHITECT system (Abbott), a semiquantitative chemiluminescent microparticle immunoassay. The assay was performed by The Doctors Laboratory. An index value cutoff of 0.8 was used to classify samples as antibody-positive (≥0.8) (refs. ^29,30^).

### Quantitative inhibition of ACE2 binding to SARS-CoV-2 spike

Quantitative inhibition of ACE2 binding to trimeric SARS-CoV-2 spike protein from VOCs was measured using the MSD V-PLEX COVID-19 ACE2 Neutralization Kit (SARS-CoV-2 plate 7) according to the manufacturer’s instructions (lot no. K0081681). Briefly, 96-well plates were blocked. After washing, samples diluted 1:10 in the diluent, as well as reference standards, were added to the plate. After incubation, SULFO-TAG Human ACE2 Protein detection protein was added to the plate and incubated for 1 h. Plates were washed before reading immediately using a MESO QuickPlex SQ 120 system. Data were generated by the Methodological Mind software and analyzed with the MSD Discovery Workbench v.4.0 software. The presented data were adjusted for any sample dilution.

### Quantification of SARS-CoV-2-specific cellular responses by ELISpot analysis

PepMix pools containing 15-mer peptides overlapping by 10 amino acids from either SARS-CoV-2 spike S1 or S2, N/M/E protein domains were purchased from Alta Biosciences (University of Birmingham). T cell responses of postvaccination samples to the above peptide mixes were determined using a Human IFN-γ ELISpot PRO kit (Mabtech). Isolated PBMCs were thawed and rested overnight before assay in R10 (RPMI 1640 + 10% FCS + penicillin/streptomycin). Then, 2–3 × 10^5^ PBMCs were stimulated in duplicate with peptide mixes at 1 μg ml^−1^ per peptide, anti-CD3 and CEFX cell stimulation mix (JPT) as a positive control or DMSO as a negative control for 16–18 h. Supernatants were collected and stored at −80 °C. After the development of plates according to the manufacturer’s instructions, plates were read using the BIOSYS Bioreader 5000. Mean spot counts in DMSO-treated negative control wells were deducted from the means to generate normalized spot counts for all other treated wells. Cutoff values were determined previously﻿^14^.

Cytokine concentrations in the ELISpot supernatants were assayed using a LEGENDplex Human Th Panel (BioLegend) according to the manufacturer’s instructions. Data were analyzed using the LEGENDplex Data Analysis Software Suite (BioLegend).

### Assessment of plasma cytokine levels

Multiplex assays to quantitatively measure levels of ten different cytokines (IFN-γ, IL-1β, IL-2, IL-4, IL-6, IL-8, IL-10, IL-12p70, IL-13 and TNF-α) in the plasma were performed using the MSD V-PLEX Proinflammatory Panel 1 (human) kit according to the manufacturer’s instructions (lot no. K0081665). Briefly, 96-well plates were washed before adding serum diluted 1:4 in diluent and calibrators. After incubation, plates were washed and detection antibodies were added. Plates were washed and read immediately using the MESO QuickPlex SQ 120 system. Data were generated by the Methodological Mind software and analyzed with the MSD Discovery Workbench v.4.0 software. The presented data were adjusted for any sample dilution.

### Cell surface staining

Cryopreserved PBMCs were thawed and rested for at least 6 h in filtered R10 medium; 10^6^+ cells were then stimulated with a combined SARS-CoV-2 spike S1 and S2 peptide pool or an N/M/E protein peptide pool at a final concentration of 1 μg ml^−1^ per peptide or DMSO for an unstimulated control. Purified anti-CD40 antibody was added to the cell suspension at final concentration 1 μg ml^−1^. Cells were incubated at 37 °C overnight for 16 h. After stimulation, cells were washed (PBS + 5% BSA + 1% EDTA), Brilliant Stain Buffer (BD) was added and cells were surface-stained at 4 °C for 30 min (Supplementary Table 2). Cells were washed and run on a BD Symphony A3 flow cytometer (BD Biosciences) and analysis was carried out using FlowJo v.10.7.1.

### Statistical analysis

All data were blinded to the investigators. Data distribution was assumed to be normal but this was not formally tested. For comparative analysis of two groups a Mann–Whitney *U*-test was applied. For comparative analysis with three or more groups, an ordinary one-way analysis of variance was used; for multiple comparisons, a Tukey’s multiple comparisons test was used. Pearson rank correlation coefficients were calculated and tested for correlations. All linear regressions were performed using the following equation: Y = a × X + b. Data analysis was performed in Prism v.9.1.0 (GraphPad Software).

### Reporting summary

Further information on research design is available in the Nature Research Reporting Summary linked to this article.

## Supplementary information

Supplementary Information Supplementary Tables 1–3.

Reporting Summary

## Data Availability

De-identified test results and limited meta-data will be made available for use by researchers in future studies, subject to appropriate research ethical approval, once the VIVALDI study cohort has been finalized. These datasets will be accessible via the Health Data Research UK Gateway (https://www.healthdatagateway.org/). All other data supporting the findings of this study are available from the corresponding author upon reasonable request.
