# Supplementary material for: Robust SARS-CoV-2-specific and heterologous immune responses in vaccine-naïve residents of long-term care facilities who survive natural infection
Source: Nat Aging. 2022 May 30;2(6):536–47. doi: 10.1038/s43587-022-00224-w (PMC10154219; doi:10.1038/s43587-022-00224-w)
Supplement: Supplementary file 1 — Supplementary Tables 1–3. [file 43587_2022_224_MOESM1_ESM.pdf]

nature portfolio

https://doi.org/10.1038/s43587-022-00224-w

Supplementary information

Robust SARS-CoV-2-specific and heterologous immune responses in vaccine-naïve residents of long-term care facilities who survive natural infection

In the format provided by the authors and unedited

Supplementary

Age group (years) and timepoint	<40 TP1	<40 TP2	<40 TP3	40-64 TP1	40-65 TP2	40-64 TP3	65-84 TP1	65-84 TP2	65-84 TP3	>85 TP1	>85 TP2	>85 TP3
**Spike-specific antibody response**												
**Number of values**	19	19	19	67	67	67	42	42	42	35	35	35
**Minimum**	191.1	86.55	99.61	388.1	31.37	31.75	62.46	17.45	49.23	64.18	32.92	49.65
**25% Percentile**	1848	3773	2982	3164	3788	3251	2913	2531	2185	1950	3057	2485
**Median**	5044	7079	5995	6670	7615	6539	6102	6519	8136	9788	12798	10419
**75% Percentile**	13568	16999	15834	18386	16959	14119	19060	18110	20998	22645	28968	23795
**Maximum**	105561	113477	82380	189014	194872	147131	146310	187957	359073	254745	249242	203003
**Range**	105370	113390	82281	188626	194840	147100	146248	187940	359024	254681	249209	202953
**Mean**	12158	14914	13327	13821	13831	12391	20570	23185	28336	23279	27096	21867
**Std. Deviation**	23334	25377	20470	24836	24851	19930	33146	39691	62677	44518	46553	39112
**Std. Error of Mean**	5353	5822	4696	3034	3036	2435	5115	6125	9671	7525	7869	6611
**Geometric mean**	4791	5712	5255	6759	6225	5687	6303	5921	6618	6122	6742	5794
**Geometric SD factor**	4.217	5.238	4.979	3.359	4.603	4.49	5.944	8.106	6.992	8.182	9.819	7.953
**RBD-specific antibody response**												
**Minimum**	200.8	70.01	88.52	132.9	27.92	42.08	57.27	61.53	71.9	66.7	21.24	36.81
**25% Percentile**	457.4	654.7	562.6	1243	1147	997.6	1011	752.1	664.9	527.9	681	656.5
**Median**	1588	2041	1456	2446	2675	2353	2181	2385	2464	3356	3925	2839
**75% Percentile**	5012	4817	4178	5446	6393	5156	6893	6121	6279	9162	11288	8220
**Maximum**	7889	10209	15719	62434	65125	51954	98386	79251	79291	63940	58235	50117
**Range**	7689	10139	15631	62301	65097	51912	98328	79190	79220	63873	58214	50080
**Mean**	2557	3398	3250	5092	5021	4435	8515	9385	7964	7220	9113	7677
**Std. Deviation**	2405	3261	3838	8693	8657	7236	17850	19050	15713	11571	13766	12123
**Std. Error of Mean**	551.8	748.2	880.5	1062	1058	884	2754	2939	2454	1956	2327	2049

**Geometric mean**	1434	1823	1677	2443	2328	2085	2449	2405	2251	2421	2842	2474
**Geometric SD factor**	3.38	3.865	3.719	3.431	3.969	3.822	5.009	5.647	5.29	5.92	6.588	5.752
**Nucleocapsid-specific antibody response**												
**Minimum**	454.8	152.1	143.7	90.23	79.61	70.33	25.22	41.99	41.21	25.22	41.99	41.21
**25% Percentile**	3353	3744	2230	6571	5706	3808	4679	3138	2964	2532	2406	1920
**Median**	12522	13399	8648	13962	12208	7398	24697	14299	11819	28699	27154	15553
**75% Percentile**	32511	40948	24910	32553	24819	18818	44674	44235	32908	55249	57078	31568
**Maximum**	81511	84452	51809	134245	164144	115855	194471	170952	110074	308046	359406	333983
**Range**	81056	84300	51665	134155	164064	115785	194445	170910	110032	308021	359364	333942
**Mean**	23219	22845	15281	26860	24302	15633	31968	28207	22497	55101	51040	33431
**Std. Deviation**	26755	26447	17037	31084	32163	20881	38607	35695	26623	80737	77902	62637
**Std. Error of Mean**	6138	6067	3909	3798	3929	2551	5957	5508	4158	13647	13168	10588
**Geometric mean**	10019	8336	5943	13503	10339	6875	13551	10726	8381	14921	13503	8603
**Geometric SD factor**	4.838	6.336	5.949	3.832	4.965	4.56	5.369	5.619	5.812	7.95	8.174	7.242

Supplementary table 1: Median, mean, and geometric mean values of spike-specific, RBD-specific and nucleocapsid-specific IgG response in SARS-CoV-2 seropositive donors. Donors are divided into four age ranges. TP1 = baseline; TP2 = 2 months follow up; TP3 = 4 months follow up.

Antigen	Clone	Fluorophore	Supplier	Code
BD Horizon Fixable Viability Dye	-	FVS575V	BD	565694
CD14	M5E2	BV650	Biolegend	301836
CD19	HIB19	BV650	Biolegend	302238
CD3	SK7	AF700	Biolegend	344822
CD8	SK1	BUV805	BD	612889
CD4	SK3	BUV496	BD	612936
CD27	L128	BUV563	BD	748705
CD25	2A3	BUV615	BD	612996
CD127	HIL-7R-M21	BUV737	BD	612794
CD45RA	HI100	BV480	BD	566114
CCR7	2-L1-A	APC-Cy7	Biolegend	353212
CD69	FN50	BV711	BD	563836
HLA-DR	G46-6	BV786	BD	564041
TCRgd	11F2	BB700	BD	745944
CD56	B159	PE-Cy5	BD	555517
CD28	CD28.2	BUV661	BD	741635
CD39	TU66	A1	Biolegend	328224
CD95	DX2	PE-Cy7	Biolegend	305622
CD154	24-31	APC	Biolegend	310810
CD137	4B4-1	PE	Biolegend	309804
Purified anti-CD40	HB14	-	Biolegend	313020

Supplementary table 2: Cell surface staining panel (AIM)

**Age quartile**	**Mean IgG N response**	
**<40** baseline	23219	
**<40** 2 months	22845	
**<40** 4 months	15281	
**% Change**	-34.187519	p=0.0390
**40-64** Baseline	26860	
**40-65** 2 **months**	24302	
**40-64** 4 **months**	15633	
**% Change**	-41.798213	p=<0.0001
**65-84** Baseline	31968	
**65-84** 2 **months**	28207	
**65-84** 4 **months**	22497	
**% Change**	-29.626502	p=0.3758
**>85** baseline	55101	
**>85** 2 **months**	51040	
**>85** 4 **months**	33431	
**% Change**	-39.32778	p=0.0003

Supplementary Table 3: Nucleocapsid-specific IgG response within age quartiles

Mean nucleocapsid-specific antibody responses at baseline and 2 and 4 month follow up within four age groups, <40, 40-64, 65-85 and >85-year-olds
