# Supplementary material for: Robust SARS-CoV-2-specific and heterologous immune responses in vaccine-naïve residents of long-term care facilities who survive natural infection
Source: Nat Aging. 2022 May 30;2(6):536–47. doi: 10.1038/s43587-022-00224-w (PMC10154219; doi:10.1038/s43587-022-00224-w)
Supplement: Supplementary file 2 — Reporting Summary [file 43587_2022_224_MOESM2_ESM.pdf]

nature portfolio

Corresponding author(s): Gokhan Tut

Last updated by author(s): March 22nd 2022

Reporting Summary

Nature Portfolio wishes to improve the reproducibility of the work that we publish. This form provides structure for consistency and transparency in reporting. For further information on Nature Portfolio policies, see our Editorial Policies and the Editorial Policy Checklist.

Statistics

For all statistical analyses, confirm that the following items are present in the figure legend, table legend, main text, or Methods section.

n/a	Confirmed	
☐	☒	The exact sample size (n) for each experimental group/condition, given as a discrete number and unit of measurement
☐	☒	A statement on whether measurements were taken from distinct samples or whether the same sample was measured repeatedly
☐	☒	The statistical test(s) used AND whether they are one- or two-sided<br>*Only common tests should be described solely by name; describe more complex techniques in the Methods section.*
☒	☐	A description of all covariates tested
☐	☒	A description of any assumptions or corrections, such as tests of normality and adjustment for multiple comparisons
☐	☒	A full description of the statistical parameters including central tendency (e.g. means) or other basic estimates (e.g. regression coefficient) AND variation (e.g. standard deviation) or associated estimates of uncertainty (e.g. confidence intervals)
☐	☒	For null hypothesis testing, the test statistic (e.g. F, t, r) with confidence intervals, effect sizes, degrees of freedom and P value noted<br>*Give P values as exact values whenever suitable.*
☒	☐	For Bayesian analysis, information on the choice of priors and Markov chain Monte Carlo settings
☒	☐	For hierarchical and complex designs, identification of the appropriate level for tests and full reporting of outcomes
☐	☒	Estimates of effect sizes (e.g. Cohen's d, Pearson's r), indicating how they were calculated

Our web collection on statistics for biologists contains articles on many of the points above.

Software and code

Policy information about availability of computer code

Data collection	All data was generated from these software: BD FACS DIVA v9.1, Methodological minds (MSD), Biosensor 500 (ELISpot) and Ledengplex analysis software (webpage)
Data analysis	All statistical analysis was done on Graphpad Prism 9 v9.1.0 and FlowJo v0.71

For manuscripts utilizing custom algorithms or software that are central to the research but not yet described in published literature, software must be made available to editors and reviewers. We strongly encourage code deposition in a community repository (e.g. GitHub). See the Nature Portfolio guidelines for submitting code & software for further information.

Data

Policy information about availability of data

All manuscripts must include a data availability statement. This statement should provide the following information, where applicable:

- Accession codes, unique identifiers, or web links for publicly available datasets
- A description of any restrictions on data availability
- For clinical datasets or third party data, please ensure that the statement adheres to our policy

De-identified test results and limited meta-data will be made available for use by researchers in future studies, subject to appropriate research ethical approvals, once the VIVALDI study cohort has been finalised. These datasets will be accessible via the Health Data Research UK Gateway https://www.healthdatagateway.org/.

Field-specific reporting

Please select the one below that is the best fit for your research. If you are not sure, read the appropriate sections before making your selection.

☒ Life sciences ☐ Behavioural & social sciences ☐ Ecological, evolutionary & environmental sciences

For a reference copy of the document with all sections, see nature.com/documents/nr-reporting-summary-flat.pdf

Life sciences study design

All studies must disclose on these points even when the disclosure is negative.

Sample size

A total of 276 participants were used in our study and this included 85 over 80 years olds, 46 between 65-80 years old and 125 under 65 year olds. Across 38 different care homes in England. This cohort represents the care home populations well in England. Samples were chosen based on the sample collection occurring for the wider VIVALDI study. No statistical analysis was done for determining sample size. This is one of the largest studies to look at natural infection in care homes.

Data exclusions

Both staff and residents were eligible for inclusion if it was possible to link them to a pseudo-identifier in the COVID-19 Datastore. Only those that had been infected in the first wave of the pandemic in the UK or those that had no evidence of being infected were included in this sub-cohort. Samples from LTCFs that had experienced outbreaks in the first wave were chosen for seropositive samples and LTCFs with no outbreaks were chosen for the seronegative samples. Participants who seroconverted during the 4-month period of follow up were not included in the analysis. Due to limited PCR testing in the first wave of the pandemic, it was not possible to determine the date of primary infection with SARS-CoV-2. 21st April 2020 was the peak of the first wave for SARS-CoV-2 infection within LTCFs. Past infection with SARS-CoV-2 was defined based on results of Abbott antibody and MSD quantitative test using thresholds and methods outlined below. Past infection with SARSCoV-2 was defined based on results of Abbott antibody and MSD quantitative test using thresholds and methods outlined below. No other data was excluded besides whats stated above

Replication

All samples we run in duplicates at least and we have more sample left if anyone wanted to re test our samples. All duplicated experiments were successful

Randomization

All donors we assigned a ID number and only information that was essential for analysis (e.g. age) was included

Blinding

All investigators we blinded to the cohort

Reporting for specific materials, systems and methods

We require information from authors about some types of materials, experimental systems and methods used in many studies. Here, indicate whether each material, system or method listed is relevant to your study. If you are not sure if a list item applies to your research, read the appropriate section before selecting a response.

Materials & experimental systems

n/a	Involved in the study	
☐	☒	Antibodies
☒	☐	Eukaryotic cell lines
☒	☐	Palaeontology and archaeology
☒	☐	Animals and other organisms
☐	☒	Human research participants
☒	☐	Clinical data
☒	☐	Dual use research of concern

Methods

n/a	Involved in the study	
☒	☐	ChIP-seq
☐	☒	Flow cytometry
☒	☐	MRI-based neuroimaging

Antibodies

Antibodies used

Antigen Clone Fluorophore Supplier Code
BD Horizon Fixable Viability Dye - FVS575V BD 565694 (1ul)
CD14 M5E2 BV650 Biolegend 301836 (2.5ul)
CD19 HIB19 BV650 Biolegend 302238 (1ul)
CD3 SK7 AF700 Biolegend 344822(1ul)
CD8 SK1 BUV805 BD 612889(1ul)
CD4 SK3 BUV496 BD 612936(1ul)
CD27 L128 BUV563 BD 748705(1ul)
CD25 2A3 BUV615 BD 612996(1ul)
CD127 HIL-7R-M21 BUV737 BD 612794(2.5ul)
CD45RA HI100 BV480 BD 566114(1ul)
CCR7 2-L1-A APC-Cy7 Biolegend 353212(5ul)
CD69 FN50 BV711 BD 563836(2.5ul)

	SULFO-TAG Anti-Human IgG Antibody - supplied from MSD with the MSD kit. used 1<br><br>HLA-DR G46-6 BV786 BD 564041 (2.5ul)<br>TCRgd 11F2 BB700 BD 745944 (2.5ul)<br>CD56 B159 PE-Cy5 BD 555517(5ul)<br>CD28 CD28.2 BUV661 BD 741635(2.5ul)<br>CD39 TU66 A1 Biolegend 328224(5ul)<br>CD95 DX2 PE-Cy7 Biolegend 305622(2.5ul)<br>CD154 24-31 APC Biolegend 310810(2.5ul)<br>CD137 4B4-1 PE Biolegend 309804(2.5ul)<br>Purified anti-CD40 HB14 - Biolegend 313020 (2.5ul)<br>SULFO-TAG Anti-Human IgG Antibody - supplied from MSD with the MSD kit. used 1:1000
Validation	All antibodies validated by the supplier, additionally all antibodies were tested and titrated using healthy donor PBMC. Data not shown.

Human research participants

Policy information about studies involving human research participants

Population characteristics	All relevant information provided in table 1
Recruitment	Sample Collection<br>The VIVALDI study (ISRCTN14447421) (ongoing) is a prospective cohort study, which was set up in May 2020 to investigate SARS-CoV-2 transmission, infection outcomes, and immunity in residents and staff in LTCFs in England that provide residential and/or nursing care for adults aged 65 years and over (https://wellcomeopenresearch.org/articles/5-232/v2; (9)). Eligible LTCFs were identified by the Care Provider's Senior Management Team, or by the National Institute for Health Research (NIHR) Clinical Research Network. Pseudonymised clinical (vaccination status, PCR test results) and demographic (age, sex, staff member versus resident) data were retrieved for staff and residents from participating LTCFs through national surveillance systems. All participants provided written informed consent for blood sample collection or if residents lacked the capacity to consent, a personal or nominated consultee was identified to act on their behalf.<br>Blood sampling was offered to participants at three-time points in June-July, August-September and October-November 2020. This time period was before the national vaccine rollout and therefore all data assess the immune response to natural infection alone. Anti-coagulated (EDTA) sample was sent to the University of Birmingham and serum tube to The Doctors Laboratory for anti-nucleocapsid IgG (N) testing.
Ethics oversight	Ethical approval for this study was obtained from the South Central - Hampshire B Research Ethics Committee, REC Ref: 20/SC/0238.

Note that full information on the approval of the study protocol must also be provided in the manuscript.

Flow Cytometry

Plots

Confirm that:

- ☒ The axis labels state the marker and fluorochrome used (e.g. CD4-FITC).
- ☒ The axis scales are clearly visible. Include numbers along axes only for bottom left plot of group (a 'group' is an analysis of identical markers).
- ☒ All plots are contour plots with outliers or pseudocolor plots.
- ☒ A numerical value for number of cells or percentage (with statistics) is provided.

Methodology

Sample preparation	Samples were processed within 24 hours of reception at the University of Birmingham. Blood was spun at 300g for 5 minutes. Plasma was removed and spun at 500g for 10 minutes prior to storage at -80°C. The remaining blood was separated using a SepMate (Stemcell) density centrifugation tube. The resulting PBMC layer was washed twice with RPMI and frozen in freezing media (10% DMSO 90% FBS).<br><br>Cryopreserved PBMC were thawed and rested for at least 6 hours in filtered R10. 106+ cells were then stimulated with a combined SARS-CoV-2 Spike S1 and S2 peptide pool or a Nucleocapsid, Envelope and Membrane protein peptide pool at final concentration of 1µg/ml per peptide, or DMSO for an unstimulated control. Purified anti-CD40 antibody was added to the cell suspension at final concentration 1µg/ml. Cells were incubated at 37oC overnight for 16 hours. Following stimulation, cells were washed (PBS+5% BSA+1% EDTA), Brilliant Stain Buffer (BD) was added and cells were surface stained at 4oC for 30 minutes (supplementary table 2).
Instrument	Cells were washed and run on a BD Symphony A3 flow cytometer (BD Biosciences)
Software	analysis was carried out using FlowJo v10.7.1

Cell population abundance	No cell sorting was done
Gating strategy	Singlets were chosen first (FSC-H vs FSC), then lymphocytes (SSC vs FSC), then live cells while removing monocytes (Live vs dump), then CD3 positive cells were isolated to get CD4 and CD8 cells, or NK cells (CD56+) or TCRgd cells (TCRgd+).<br>Gating strategy available in supplementary figure 1

☒ Tick this box to confirm that a figure exemplifying the gating strategy is provided in the Supplementary Information.
